# Different chemical proteomic approaches to identify the targets of lapatinib

**DOI:** 10.1080/14756366.2023.2183809

**Published:** 2023-03-01

**Authors:** Tatjana Kovačević, Krunoslav Nujić, Mario Cindrić, Snježana Dragojević, Adrijana Vinter, Amela Hozić, Milan Mesić

**Affiliations:** a Selvita Ltd., Zagreb, Croatia; b Ruđer Bošković Institute, Zagreb, Croatia

**Keywords:** Chemical proteomics, affinity chromatography, diazirines, photoaffinity labelling, EGFR, lapatinib, protein identification

## Abstract

The process of identifying the protein targets and off-targets of a biologically active compound is of great importance in modern drug discovery. Various chemical proteomics approaches have been established for this purpose. To compare the different approaches, and to understand which method would provide the best results, we have chosen the EGFR inhibitor lapatinib as an example molecule. Lapatinib derivatives were designed using linkers with motifs, including amino (amidation), alkyne (click chemistry) and the diazirine group (photo-affinity). These modified lapatinib analogues were validated for their ability to inhibit EGFR activity *in vitro* and were shown to pull down purified recombinant EGFR protein. In all of the approaches evaluated here, we identified EGFR as the main protein target from the lysate of immortalised cell line expressing EGFR, thus validating its potential use to identify unknown protein targets. Taken together, the results reported here give insight into the cellular activities of lapatinib.

## Introduction

Understanding the mode of action of potential drugs is an important task in the drug discovery process. Identification of protein targets and off-targets is vital in phenotypic-driven drug discovery projects. In a target-based project, where the protein target is defined at the beginning of the project, information about additional off-targets can also be valuable (e.g. regarding selectivity issues, toxic effects or new indications).^1^

Chemical proteomic approaches for identification of the protein targets of bioactive small molecules have been reviewed elsewhere.^2–6^ The main advantage of the chemical proteomics approach is the isolation and identification of proteins targets from the cellular environment, in which protein conformations, post-translational modifications and protein complexes are preserved. The bioactive small molecules were derivatized by introducing a reactive group via various linkers, followed by their immobilisation onto a solid support. Primary amines, alkynes and photoreactive group^7–9^ are groups that are frequently used for derivatization of bioactive molecules, using linkers of various structures and lengths. Solid supports also vary, with sepharose- and agarose-based polymers (resins) being the matrices used most often. Typically, protein mixtures, obtained from cell lysates or tissue homogenates, are incubated with immobilised ligands on these resins. Proteins that interact with the ligand-matrix can then be subjected to either an in-solution sequence-specific protease digestion or separated by electrophoresis, followed by in-gel sequence-specific protease digestion. The digests are then submitted for mass spectrometry analysis, to support identification of potential targets and off-targets. These proteins can then be validated through specific biochemical or cellular assays.

Although these commonly used methods and technologies provide valuable information about protein targets, they also have some limitations and display experimental variability, thus preventing comprehensive and unambiguous determination of compound-protein interactions. Factors that increase the degree of variability in chemical proteomics^10^ include: the positions of the linker on the small molecule (ligand), the source of protein (cell or tissue type), details of sample preparation for mass spectrometry and the type of mass spectrometry used for protein identifications. Additionally, all these approaches also have common limitations such as structural modification of bioactive molecule or non-specific binding to linkers and matrices, as well as difficulties in the identification of low expression or membrane proteins. Due to these limitations and other sources of variability, target identification experiments with identical or similar bioactive molecules can give substantially different results, depending on conditions used at various stages of the process.

To investigate some of these limitations and to obtain a more comprehensive data set, we performed target identification of a bioactive molecule using a combination of three different reactive groups, two linkers and two solid supports. As a case study, we have selected lapatinib (**1**), a dual inhibitor of epidermal growth factor receptors (EGFR/ErbB-1 and HER2/ErbB-2). The EGFR and HER2 proteins are receptor tyrosine kinases whose dysregulation has been associated with a number of cancers.^11^^,^^12^ The binding mode of lapatinib in the ATP binding pocket is common and therefore potential interaction with other kinases could be possible. In addition, interactions with other protein families are also plausible. For example, it was confirmed that lapatinib interacts with the protein disulphide isomerase (PDI) using ligand-directed tosylation (LDT) chemistry under live cell conditions.^13^

## Results

### Docking studies

**Figure 1.**
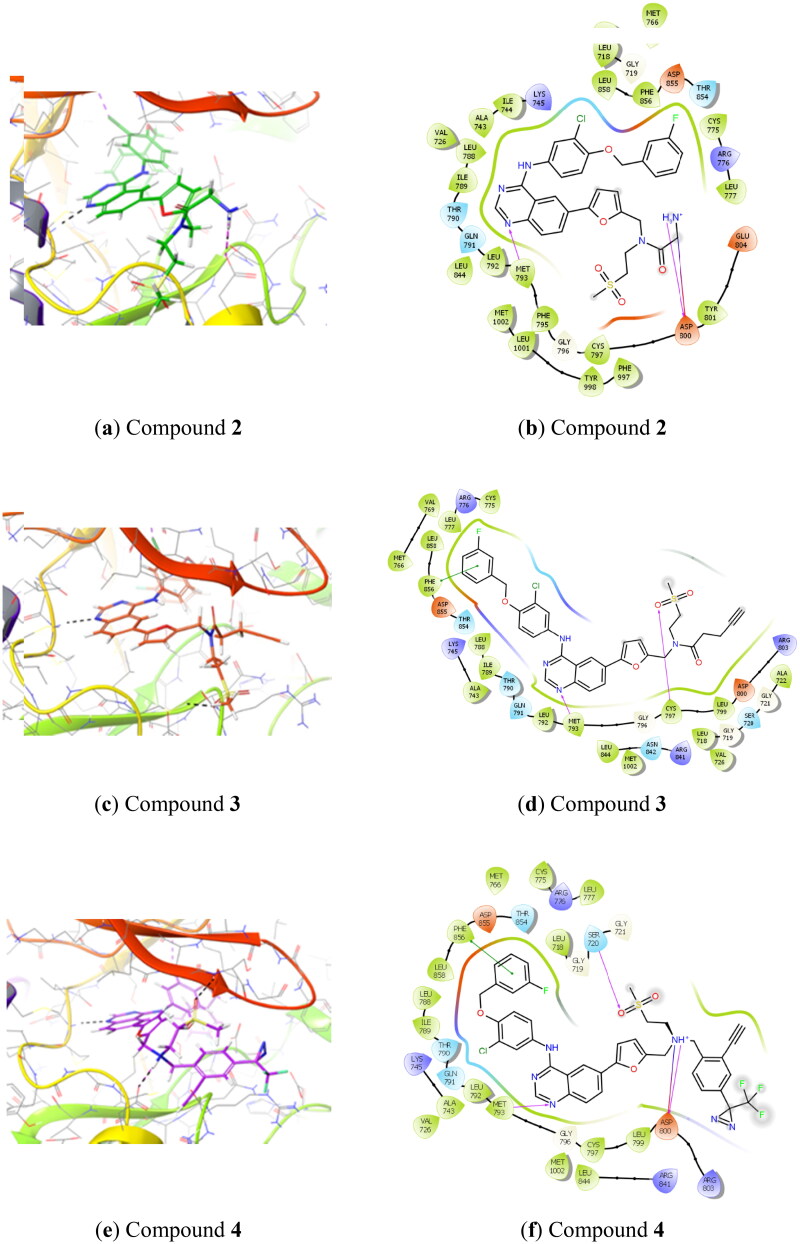
Predicted binding of lapatinib analogues to the EGFR receptor site (docked to PDB: 1xkk) (1a, 1c, 1e) and corresponding schematic diagrams (1b, 1d, 1f).

Based on protein-ligand X-ray crystallography data of lapatinib bound to the EGFR kinase domain (PDB: 1xkk)^14^ we performed computational docking of proposed lapatinib analogues using Glide from the Schrodinger software suite (Figure 1). This demonstrated that all of the proposed lapatinib analogues could bind to EGFR, preserving the original lapatinib binding mode. The compounds **2** and **3** (amine and alkyne bearing analogues) are oriented towards the solvent exposed region of EGFR (Figures 1(a–d)) and we do not expect that they will interfere significantly with the protein. Also, compound **4,** which carries a large phenyl group onto which are attached dual tag motifs (diazirine and alkyne at the phenyl ring), could possibly by accommodated in the “solvent-exposed” region (Figures 1(e,f)). Nevertheless, we anticipated that the largest influence on *in vitro* affinity towards EGFR would be from compound **4**.

### Synthesis

**Scheme 1.**
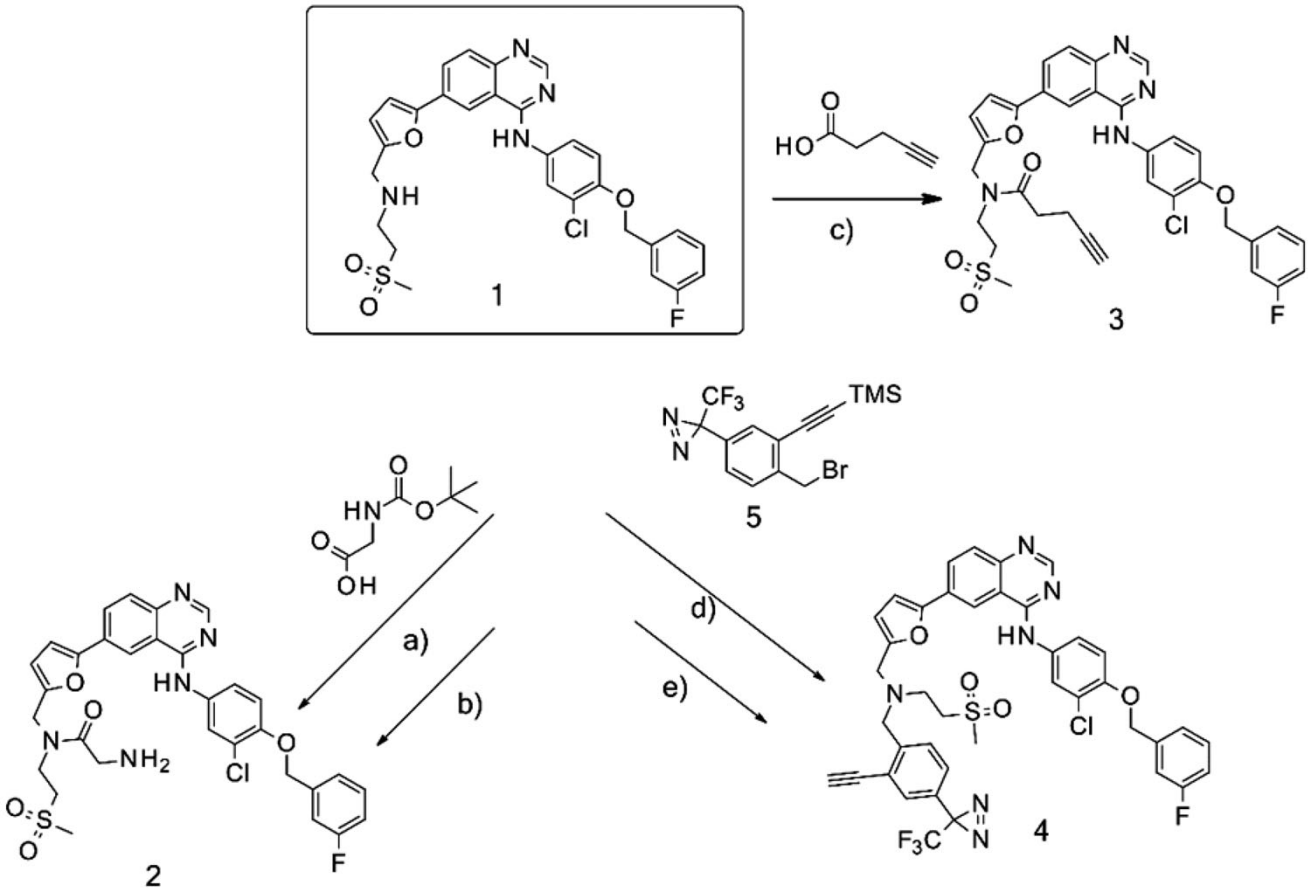
Synthetic approach to synthesised analogues of lapatinib. Reagents and conditions: (a) PS-CDI, HOBT, DIPEA, DCM, DMF, rt; (b) TFA, DCM, rt; (c) PS-CDI, HOBT, DIPEA, DCM, DMF, rt; (d) NaH, DMF, rt; (e) K_2_CO_3_, MeOH, rt.

The synthesis of the lapatinib analogues was achieved starting from **1** and yielded molecules **2**–**4**. All transformations were focused on the secondary amine functionality of **1**. These modifications (amidation) changed secondary amino group from basic to neutral in the case of compounds **2** and **3**, while for molecule **4** an alkylation reaction preserved the basicity of this centre. The synthesis of molecules **2**–**4** was accomplished using the approach described in Scheme 1, in each one or two synthesis reaction steps. To access compound **2**, amidation of lapatinib **1** was performed with 2-(*tert*-butoxycarbonylamino)acetic acid using polymer-supported carbodiimide (PS-CDI), hydroxybenzotriazole (HOBT) and *N,N*-diisopropylethylamine (DIPEA) in DCM/DMF at room temperature. After isolation and purification of the protected intermediate, *tert*-butoxycarbonyl protecting group (BOC) was removed using trifluoroacetic acid (TFA) in DCM at room temperature. The final molecule **2** was purified by flash chromatography on prepacked silica columns. Similarly, we used the same chemistry to amidate compound **1** with propargylacetic acid, to yield compound **3**. To prepare lapatinib with a dual tag functionality (**4**), we initially needed to prepare the bromobenzyl intermediate **5**.

**Scheme 2.**
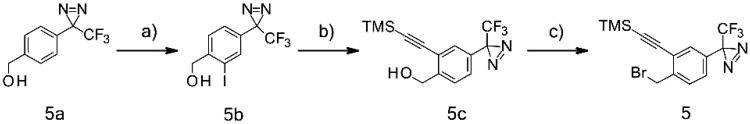
Synthesis of intermediate **5**. Reagents and conditions: (a) thallium(III) trifluoroacetate, TFA, NaI, CF_3_SO_3_H, H_2_O, 80 °C, 2h; (b) (trimethylsilyl)acetylene, CuI, TEA, PdCl_2_(PPh_3_)_2_, THF, rt, on; (c) Ph_3_P, CBr_4_, DCM, rt, on.

Intermediate **5** was synthesised from commercially available [4-[3-(trifluoromethyl)diazirin-3-yl]phenyl]methanol **5a** (Scheme 2). Iodination of **5a** was accomplished by reaction of thalium(III) trifluoroacetate and trifluoromethane-sulfonic acid in trifluoroacetic acid/water solution, followed by the addition of sodium iodide providing **5b**. A trimethylsilyl (TMS) protected alkyne was then cross-coupled onto **5b** using the Sonogashira reaction to afford **5c**. Finally, benzylic alcohol was converted to desired bromide **5** using triphenylphosphine and tetrabromomethane in DCM (Scheme 2). After alkylation of **1** with the intermediate **5**, the trimethylsilyl protecting group was removed using potassium carbonate in MeOH. Purification of **4** was performed using MS directed liquid chromatography.

### In vitro

In order to evaluate our prediction that lapatinib modifications may affect the binding to EGFR, we tested *in vitro* binding of **1** and three modified analogues of it, **2–4**. The *in vitro* IC_50_ results for all three compounds showed a slightly lower activity (2–10 fold, Table 1) than lapatinib, but were still comparable to it. As we expected from docking experiments, the highest loss in potency was observed with the dual tag functionality (**4**), due to the lack of ability of this motif to accommodate itself perfectly within the exit area of the binding pocket. Nevertheless, the loss in potency of one log unit was, in our opinion, not significant and we believed that this compound could still be valuable in the identification of the EGFR protein and possible lapatinib off-targets.

**Table 1.** *In vitro* activity of lapatinib and its analogues as EGFR inhibitors.

Compound	Structure	IC_50_ (nM)
1	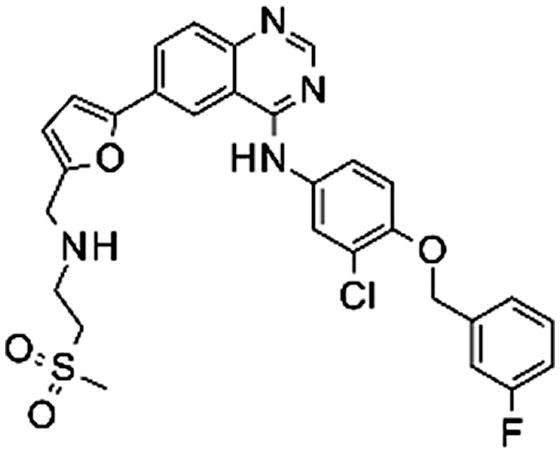	0.83
2	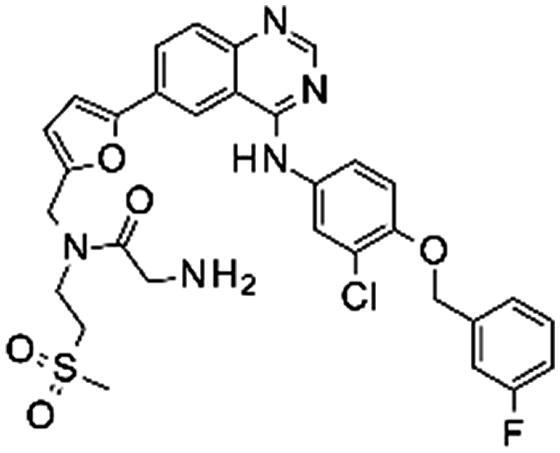	1.60
3	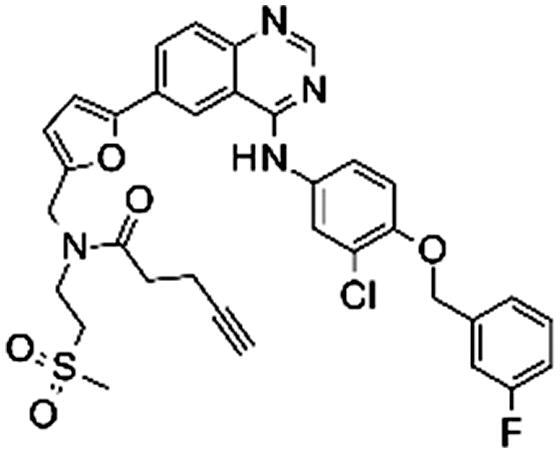	3.60
4	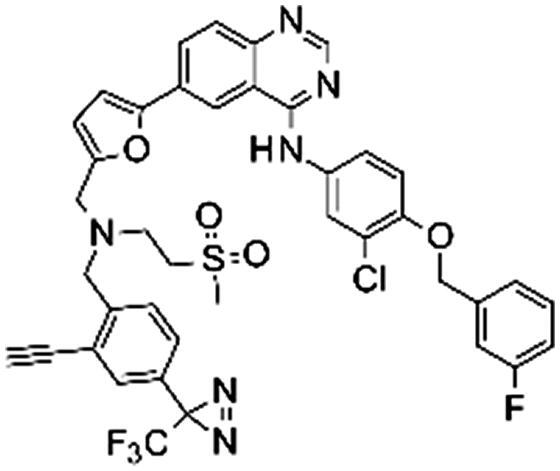	7.80

Since the modifications of lapatinib had no significant effect on activity, the analogues were further validated in target identification experiments using recombinant EGFR. We immobilised analogues **2** and **3** onto a solid support and incubated them with purified recombinant EGFR. The chemical reactions were monitored by HPLC-MS, with consumption of the compounds (**2** and **3**) seen to be approximately 90% (data not shown). We used solid support resins lacking the immobilised compound as a control for non-specific binding.

**Figure 2.**
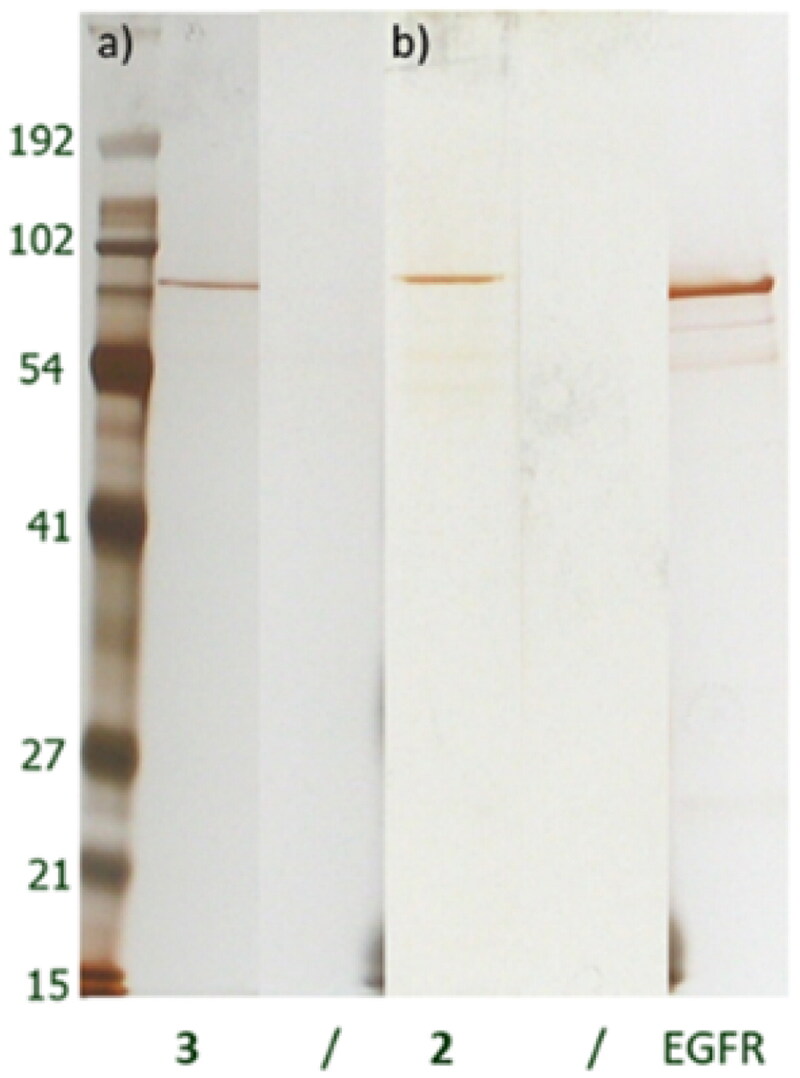
Silver-stained gels after interaction of purified recombinant EGFR with immobilised lapatinib derivatives 3 (a) and 2 (b). Matrices alone were used as negative controls (/). Purified recombinant EGFR was used as additional control.

Proteins that bound to either the amino (Figure 2, gel b) or alkyne derivative (Figure 2, gel a) were visualised by gel electrophoresis followed by silver staining. Analogue **4** was incubated with purified recombinant EGFR and then UV illumination at 320 nm was used to activate the diazirine functionality and allow reactive carbene species to react with its proximal amino acid residues. Following this, the resin was washed out. Due to the presence of a covalent bond between **4** and the protein, the gel electrophoresis step was omitted and instead in-solution tryptic digestion and mass spectrometry (ESI-MS) were performed. EGFR was identified successfully.

Having validated our approach with purified recombinant EGFR protein, we next performed a target identification experiment using A431 cell lysate. A431 cells were chosen as they have been reported ^15^^,^^16^ to express high amounts of the EGFR protein and therefore the amount of the target protein should not be the limiting factor for target identification.

**Figure 3.**
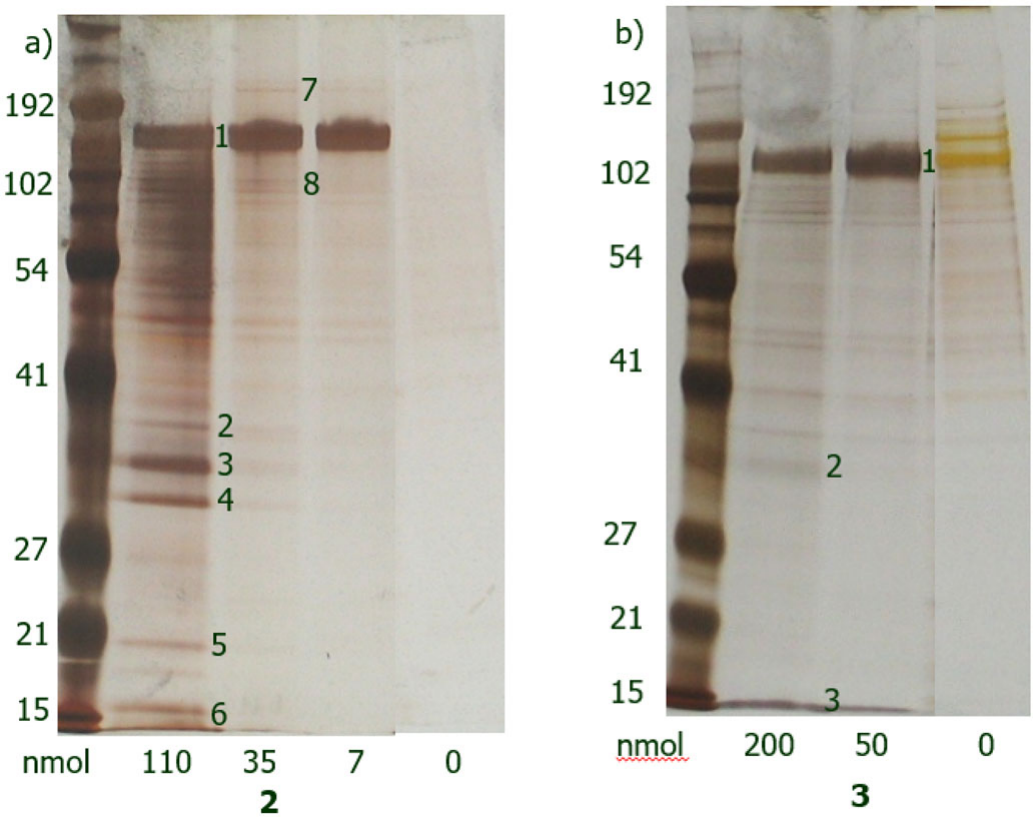
Silver stained gels of bound proteins after incubation of 2 (a) and 3 (b) derivatives with A431 cell lysate.

Ligand density within the cell can be one of the most important parameters in chemical proteomics studies. Analogues **2** and **3** were thus immobilised at a series of different concentrations onto a solid support, in order to cover a wide range of ligand densities and get a more comprehensive data set (Figures 3(a,b)).

Protein bands 1–8 were analysed by mass spectrometry (ESI-MS, Table 2 for analogue **2** and Table 3 for analogue **3**). Five of them were successfully identified for analogue **2** and one for analogue **3**. At lower ligand densities, only a single strong band was present, which was identified to be EGFR, suggesting that this protein has the strongest affinity for the immobilised compounds. Other proteins were only detected at higher ligand densities, suggesting weaker affinity for the immobilised compounds.

**Table 2.** Identified proteins captured by **2**, as identified using ESI-MS.

Band	Name	Accession no.
1	Epidermal growth factor receptor	P00533
2	Glyceraldehyde-3-phosphate dehydrogenase	P04406
3	Prohibitin 2	Q99623
4	Prohibitin	P35232
5, 7, 8	ND	ND^a^
6	Keratin, type I cytoskeletal 10	P13645

^a^ND: not determined.

**Table 3.** Identified proteins captured by **3**, as identified using ESI-MS.

Band	Name	Accession no.
1	Epidermal growth factor receptor	P00533
2, 3	ND	ND^a^

^a^ND: not determined.

Proteins from A431 cell lysate covalently bound to compound **4** after photochemical activation of the diazirine group were then identified by mass spectrometry (SYNAPT G2-Si mass spectrometer – A list of proteins that were enriched in affinity matrices relative to negative resin). Again, EGFR was identified as a protein that interacts with the lapatinib derivate, together with a few other proteins (Table 4, 6 additional proteins were identified).

**Table 4.** Identified proteins captured by **4**, as identified using ESI-MS.

	Name	Accession no.
1	Epidermal growth factor receptor	P00533
2	Fatty acid synthase	P49327
3	Protein disulphide isomerase	P07237
4	Peroxiredoxin 1	Q06830
5	Pyruvate kinase PKM	P14618
6	Histone H4	P62805
7	40S ribosomal protein S5	P46782

In summary, EGFR was confirmed as the main target of lapatinib in A431 cells regardless of the analogue used. Other proteins identified as potential new lapatinib-interacting proteins are presented in Table 5.

**Table 5.** Identified lapatinib bound proteins across different proteomics technologies.

	Name	Accession no.
1	Epidermal growth factor receptor	P00533
2	Protein disulphide isomerase	P07237
3	Peroxiredoxin 1	Q06830
4	Prohibitin	P35232
5	Prohibitin 2	Q99623
6	Glyceraldehyde-3-phosphate dehydrogenase	P04406
7	Fatty acid synthase	P49327
8	Pyruvate kinase PKM	P14618
9	Histone H4	P62805
10	40S ribosomal protein S5	P46782

## Discussion

As described previously,^2–6^ chemical proteomics approaches have often been used as a powerful platform for identifying the protein targets of biologically active compounds. However, unavoidable limitations, as well as high variability in the possible experimental conditions, can influence the final outcome and results should be confirmed by additional experiments.^17^ To get more reliable and comprehensive results, as well as avoid some of these limitations, we used a combination of three different reactive groups and two linkers followed by trypsin digestion protocols, with lapatinib as our example compound. The synthesised lapatinib analogues, for use in a target identification experiment, were immobilised using different resins and linkers (*N*-hydroxysuccinimide activated agarose and azide agarose) and then tested on A431 cell lysate.

Using high affinity lapatinib analogues and optimised experimental conditions, we performed pull down experiments with cell lysate. All three approaches reproducibly identified EGFR as the main protein target of lapatinib. However, there were some differences between the outcomes from each method. Using alkyne and amino analogues 2 and 3 at a lower ligand density led to only one protein band being detected (EGFR) suggesting that EGFR has the highest affinity towards immobilised compounds. At higher ligand densities, other protein bands were visible, suggesting these ligands also bound additional proteins, but at lower affinities than EGFR. It is also possible that some of those identified proteins were non-specifically bound to affinity matrices (sticky proteins) or were part of a protein complex (indirect binders). The benefit of using an electrophoresis step for analogues 2 and 3 is that it facilitates approximate estimation of the binding affinities of each protein towards immobilised the ligand. In addition, determining the molecular weights of proteins by gel electrophoresis can be helpful tool for mass spectrometry identification. However, the sensitivity of gel staining is a limiting step in the overall sensitivity of the method and consequently we identified fewer proteins by this approach, when compared to the method with a dual tag analogue (gel free system). In addition, there is a greater chance of identifying indirect binders in systems in which no covalent bonds exist between the ligand and protein. Also, it should be taken into account that concentrations of the proteins do not necessarily increase over higher ligand density because of the possibility that these proteins are lower in abundance in the cell lines used. On the other hand, by using a dual tag we were able to identify a greater number of bound proteins due to the better sensitivity, at the cost of not gaining the information available from gel electrophoresis (molecular weight, band intensity and potential affinity). Using only a dual tag approach it was difficult to identify which of the proteins is the most probable target of lapatinib.

Having data from all three, approaches allowed easier identification of the protein target and provided comprehensive results about other potential binders. Due to the limitations of each approach, there was a low degree of overlap regarding the proteins identified and the final list of potential binders to lapatinib. Besides EGFR, the following proteins were identified: fatty acid synthase, pyruvate kinase PKM, histone H4, 40S ribosomal protein S5, protein disulphide isomerase, peroxiredoxin, prohibitin, prohibitin 2 and glyceraldehyde-3-phosphate dehydrogenase. Of these, protein disulphide isomerase (PDI) had been identified previously as an off-target of lapatinib. We identified PDI only with the dual tag approach, in which we had covalent bond between compound and protein. This was similar to the ligand-directed tosyl (LDT) approach performed by Itaru Hamachi and colleagues.^13^^,^^17^ Identification of PDI, in addition to EGFR, provides additional proof of the reliability of the approaches we have used. For other proteins, we could not find any previous published data about their interaction with lapatinib, suggesting them to be potential novel off-targets of lapatinib. It should be noted that glyceraldehyde-3-phosphate dehydrogenase, histone H4, 40S ribosomal protein S5 and prohibitin were also identified in other target identification studies with different compounds (internal data), which could imply potential non-specific binding to affinity resins. In the further, we plan to test and validate fatty acid synthase, pyruvate kinase PKM and peroxiredoxin as potential lapatinib off-targets using the drug affinity responsive target stability (DARTS) determination method. When combined with liquid chromatography/tandem mass spectrometry, DARTS enables the identification of proteins that bind to drug molecules that leads to a conformational change in the target protein(s).^18^^,^^19^ With this additional complementary method, the biologically relevant target proteins that bind to lapatinib can be confirmed and validated.

In conclusion, it would appear that each of the approaches used has its own limitations and display experimental variability. The photoaffinity approach was identified as the most direct and applicable. Consequently, it would be primary used for identification of the unknown targets whenever that approach is possible. Because of the lack of understanding the crystal structures of the new targets (and docking experiments), few analogues of the ligand modified with the photoaffinity group on different positions should be synthesised for target identification in that case. After identification, the biologically relevant target proteins should be confirmed and validated for each identified protein with unmodified ligand

## Materials and methods

### Characterisation of lapatinib derivatives

Chemicals and solvents were purchased from commercial sources where available and used without further purification.

The reaction progress and purity of the products were monitored by thin layer chromatography on Merck Silica gel 60 F254 aluminium plates. The stains were detected with UV light at a wavelength of 254 nm and/or 365 nm. In some cases, ultra-performance liquid chromatography was performed on a Waters Acquity UPLC instrument using a Waters Acquity UPLC C18 (2.1 × 50 mm, 1.7 μm) column. The column eluent was analysed using a Waters SQ mass spectrometer with ESI scanning in both positive and negative ion modes, from 100 to 2000 Da.

The purification of products was accomplished using normal phase chromatography with Biotage SP1® systems pre-packed silica cartridges from Biotage, or using Interchim filled silica gel with spherical particles with size 15, 25, and 50 μm (high performance and high capacity).

Purity of the compounds was determined on an HPLC-UV system Waters 2690 using a Waters Acquity UPLC C18 (2.1 × 50 mm, 1.7 μm) column.

^1^H NMR spectra were recorded on a Bruker Avance DPX 300 spectrometer at 300 MHz and on a Bruker Avance DRX 400 spectrometer at 400 MHz, in the solvent indicated. Chemical shifts are reported in parts per million (ppm) and were measured relative to TMS. Data for 1H NMR were described as: chemical shift (δ in ppm), multiplicity (s: singlet; d: doublet; t: triplet; q: quartette; m: multiplet; br: broad signal), integration, coupling constant J (Hz).

HRMS analyses were performed on an Agilent 6540 QTOF instrument equipped with an Agilent 1290 Infinity UHPLC, using a Waters XBridge column, C18 2.1 mm ID × 100 mm, 1.7 µm particle size. The mobile phase flow rate was 0.35 ml/min. Data were collected for positive and negative ions separately within a 150 to 1500 *m/z* mass range. Data acquisition and processing were performed with MassHunter software (Agilent Technology Ltd.). Mass spectrometer calibration was performed on a daily basis, according to the manufacturer’s protocol, for positive and negative ions.

### Protein identification by ESI-MS^E^

#### Instrumentation

The LC system used for sample separation and elution was a nanoACQUITY UPLC® (Waters, Milford, MA, USA) equipped with a trapping column nanoACQUITY UPLC® 2 G-V/M Symmetry® C18 Trap Column, 100 Å, 5 μm, 180 μm × 20 mm (Waters, Milford, MA, USA) and an analytical column ACQUITY UPLC® BEH130 C18, 130 Å, 1.7 μm, 100 μm × 100 mm Column (Waters, Milford, MA, USA). Trapping conditions were isocratic delivery of aqueous 0.1% formic acid, at 15 μL/min for two minutes at 40 °C. Sample separation was achieved on an analytical column at 1 μL/min by gradient elution (0.1–99% solvent B in 75 min) of channel A and B (aqueous 0.1% formic acid and 0.1% formic acid in 95% acetonitrile). Sample injection volume was 4 μL. The MS system used for protein identification was a SYNAPT G2-Si mass spectrometer (Waters, Milford, MA, USA) at a mass range of 50–4000 *m/z*. MS^E^ data were acquired in positive ion mode for all samples and with the collision cell energy alternating between low energy (4 eV) to collect peptide precursor (MS) data, and elevated energy (rising gradient from 20 to 40 eV) to obtain peptide fragmentation (MS^E^) data (standard MS^E^ procedure).

#### Data processing

The data acquired were processed using ProteinLynx Global Server software (PLGS; v. 3.0.1, Waters). Peak lists were generated after deisotoping and deconvolution. Separate databases (Human NCBI*nr*) were then created and the data were searched with trypsin as a digestion reagent and three potential miscleavages. Peptide and fragment tolerance were set to “automatic”. Oxidation M, dehydratation ST and deamidation N were allowed as variable modifications in all protein datasets.

### Synthesis

#### Synthesis of 2-amino-N-[[5-[4-[3-chloro-4-[(3-fluorophenyl)methoxy]anilino]quinazolin-6-yl]-2-furyl]methyl]-N-(2-methylsulfonylethyl)acetamide (2)

**Scheme 3.**
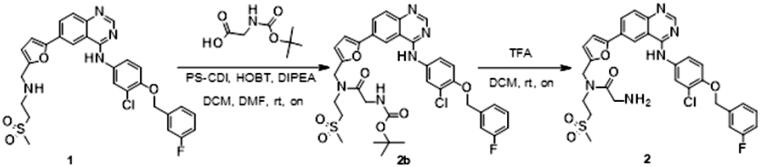
Synthesis of analogue 2. Reagents and conditions: (a) PS-CDI, HOBT, DIPEA, DCM, DMF, rt; (b) TFA, DCM, rt.

[3-Chloro-4–(3-fluoro-benzyloxy)-phenyl]-(6-{5-[(2-methanesulfonyl-ethylamino)-methyl]-furan-2-yl}-quinazolin-4-yl)-amine (**1**) (100 mg, 0.172 mmol) was added to a suspension of 2-(*tert*-butoxycarbonylamino)acetic acid (31.6 mg, 0.180 mmol), PS-Carbodiimide resin (load = 1.33 mmol/g) (168.1 mg, 0.223 mmol) HOBT hydrate (18.4 mg, 0.120 mmol) and N,N-Diisopropylethylamine (59.9 µL, 0.344 mmol) in DCM (4 ml) and DMF (0.4 ml). The reaction mixture was stirred at 25 °C overnight (Scheme 3).

Methanol (5 ml) was added and the mixture was filtered over a cotton pad. The solvent was evaporated and 5 ml of saturated NaHCO_3_ solution was added to brown oil. Extraction was then performed with EtOAc. After drying over anhydrous sodium sulphate, the solvent was evaporated to obtain a crude product. The sample was purified by flush chromatography using a BIOTAGE SP1 purification device, and a 10 g normal phase silica SNAP column (the solvent system was DCM-MeOH with gradient 0–5% of MeOH over 15 column volumes – CV). After evaporation of the solvent, 77.1 mg of *tert*-butyl N-[2-[[5-[4-[3-chloro-4-[(3-fluorophenyl)methoxy]anilino]quinazolin-6-yl]-2-furyl]methyl-(2-methylsulfonylethyl)amino]-2-oxo-ethyl]carbamate (**2b**) was isolated as a yellow solid (yield = 60.7%). and used as crude for the next reaction.

MS (Q-TOF MS): *m/z* calc. for C_36_H_37_ClFN_5_O_7_S: 738,2159 [M + H]^+^, found 738.2165 [M + H]^+^.

^1^H NMR (600 MHz, DMSO-d_6_) *δ*/ppm: 9.85 (br. s, 1H), 8.76 (s, 1H), 8.56 (s, 1H), 8.03 (s, 1H), 7.71–7.81 (m, 2H), 7.46–7.50 (m, 1H), 7.27–7.35 (m, 3H), 7.19 (t, *J* = 8.70 Hz, 1H), 7.06–7.11 (m, 1H), 6.89–6.98 (m, 1H), 6.52–6.68 (m, 1H), 5.27 (s, 2H), 4.71 (s, 1H), 4.69 (s, 1H), 4.08 (s, 1H), 3.71–3.82 (m, 2H), 3.06 (s, 1H), 3.02 (s, 2H), 2.51–2.53 (m, 1H), 1.39 (s, 3H), 1.38 (s, 6H).

Trifluoroacetic acid (238 µL, 3.09 mmol) was added to a solution of **2b** (76 mg, 0.103 mmol) in DCM (1 ml). The resulting solution was stirred at room temperature overnight. The mixture was concentrated *in vacuo*. The sample was loaded onto an SCX column (400 mg, 0.6 mmol/g, preconditioned with 25 ml of MeOH) in a mixture of DCM and MeOH. MeOH (15 ml) was passed through the column and the compound was eluted with 1.7 N NH_3_ in MeOH (15 ml). The filtrate was concentrated *in vacuo* to give 52 mg of 2-amino-N-[[5-[4-[3-chloro-4-[(3-fluorophenyl)methoxy]anilino]quinazolin-6-yl]-2-furyl]methyl]-N-(2-methylsulfonylethyl) acetamide **(2)** as a yellow solid (yield = 79%).

MS (Q-TOF MS): *m/z* calc. for C31H29ClFN5O5S 638.1635 [M + H]^+^ was found to be 638.1638.

Retention time: 9.37 min; Purity >99% (based on HPLC analysis at 230 nm).

^1^H NMR (400 MHz, DMSO-d_6_) *δ*/ppm: 9.88 (br. s, 1H), 8.73 (s, 1H), 8.57 (s, 1H), 8.10–8.18 (m, 1H), 8.03 (s, 1H), 7.71–7.84 (m, 2H), 7.44–7.51 (m, 1H), 7.27–7.36 (m, 3H), 7.15–7.22 (m, 1H), 7.05–7.11 (m, 1H), 6.52–6.66 (m, 1H), 5.27 (s, 2H), 4.70 (s, 1H), 4.67 (s, 1H), 3.71–3.81 (m, 2H), 3.50–3.60 (m, 2H), 3.34–3.40 (m, 2H), 3.04 (s, 1H).

^13^C NMR (100 MHz, DMSO-d_6_) *δ*/ppm: 163.40, 161.22, 157.56, 154.39, 152.47, 151.10, 149.77, 148.99, 139.79 (d, *J*_C-F_ = 7.45 Hz), 132.99, 130.55 (d, *J*_C-F_ = 8.76 Hz), 128.52, 127.85, 124.25, 123.34, 122.44, 121.05, 116.85, 115.29, 114.68 (d, *J*_C-F_ = 20.57 Hz), 114.33, 114.02 (d, *J*_C-F_ = 22.04 Hz), 110.93, 107.88, 69.37, 51.19, 43.41, 42.87, 42.52, 40.61.

#### Synthesis of N-[[5-[4-[3-chloro-4-[(3-fluorophenyl)methoxy]anilino]quinazolin-6-yl]-2-furyl]methyl]-N-(2-methylsulfonylethyl)pent-4-ynamide (3)

**Scheme 4.**
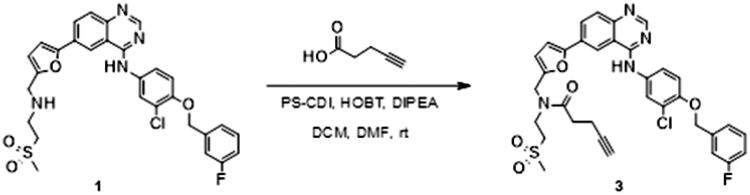
Synthesis of analogue **3.** Reagents and conditions: (a) PS-CDI, HOBT, DIPEA, DCM, DMF, rt.

N-[3-chloro-4-[(3-fluorophenyl)methoxy]phenyl]-6-[5-[(2-methylsulfonylethylamino)methyl]-2-furyl]quinazolin-4-amine (**1**), (120 mg, 0.206 mmol) was added to a suspension of pent-4-ynoic acid (20.3 mg, 0.206 mmol), PS-Carbodiimide resin (loading = 1.33 mmol/g) (201.4 mg, 0.268 mmol), HOBT hydrate (20.1 mg, 0.144 mmol) and DIPEA (107.6 µL, 0.618 mmol) in DCM (4 ml) and DMF (0.4 ml). The reaction mixture was stirred at room temperature overnight (Scheme 4).

5 ml of methanol was added and the mixture was filtered over a cotton pad. The solvent was then evaporated under reduced pressure. NaHCO_3_ saturated water solution was added to the remainder, followed by extraction with EtOAc. The solvent was evaporated to obtain a raw product.

The sample was purified by flush chromatography using a BIOTAGE SP1 purification device and 10 g normal phase silica SNAP (the solvent system was DCM-MeOH, with gradient rising from 0–5% of MeOH over 15 column volumes). After evaporation of the solvent, 77 mg of product as yellow solid was isolated (yield = 56%).

MS (Q-TOF): *m/z* calculated for C34H30ClFN4O5S 661.1682 [M + H]^+^, and found to be 661.1687.

Retention time: 10.96 min; Purity >95% (based on HPLC analysis at 230 nm).

^1^H NMR (500 MHz, DMSO-d_6_) *δ*/ppm: 9.87 (s, 1H), 8.74 (d, *J* = 12.2 Hz, 1H), 8.57 (d, *J* = 6.86 Hz, 1H), 8.12–8.18 (m, 1H), 8.00–8.06 (m, 1H), 7.79–7.84 (m, 1H), 7.72–7.77 (m, 1H), 7.45–7.51 (m, 1H), 7.27–7.36 (m, 3H), 7.19 (td, *J* = 8.39 Hz, 2.67 Hz, 1H), 7.08 (dd, *J* = 20.22 Hz, 3.05 Hz, 1H), 6.68 (dd, *J* = 65.20 Hz, 3.05Hz, 1H), 5.27 (s, 2H), 4.75 (s, 1H), 4.69 (s, 1H), 3.74–3.85 (m, 2H), 3.36 (m, 2H), 3.05 (s, 1H), 3.03 (s, 1H), 2.72–2.82 (m, 3H), 2.40–2.48 (m, 2H).

^13^C NMR (125 MHz, DMSO-d_6_) *δ*/ppm: 163.00, 161.38, 157.55, 154.39, 152.50, 151.18, 149.78, 148.96, 139.64 (d, *J*_C-F_ = 7.20 Hz), 133.01, 130.55 (d, *J*_C-F_ = 8.34 Hz), 128.52, 127.87, 124.19, 123.33, 122.38, 121.06, 116.90, 115.29, 114.68 (d, *J*_C-F_ = 20.57 Hz), 114.32, 114.03 (d, *J*_C-F_ = 21.36 Hz), 110.85, 107.80, 71.15, 69.40, 51.26, 44.41, 41.33, 40.61, 31.60, 13.79.

#### Synthesis of N-[[5-[4-[3-chloro-4-[(3-fluorophenyl)methoxy]anilino]quinazolin-6-yl]-2-furyl]methyl]-N-(2-methylsulfonylethyl)pent-4-ynamide (4)

**Scheme 5.**
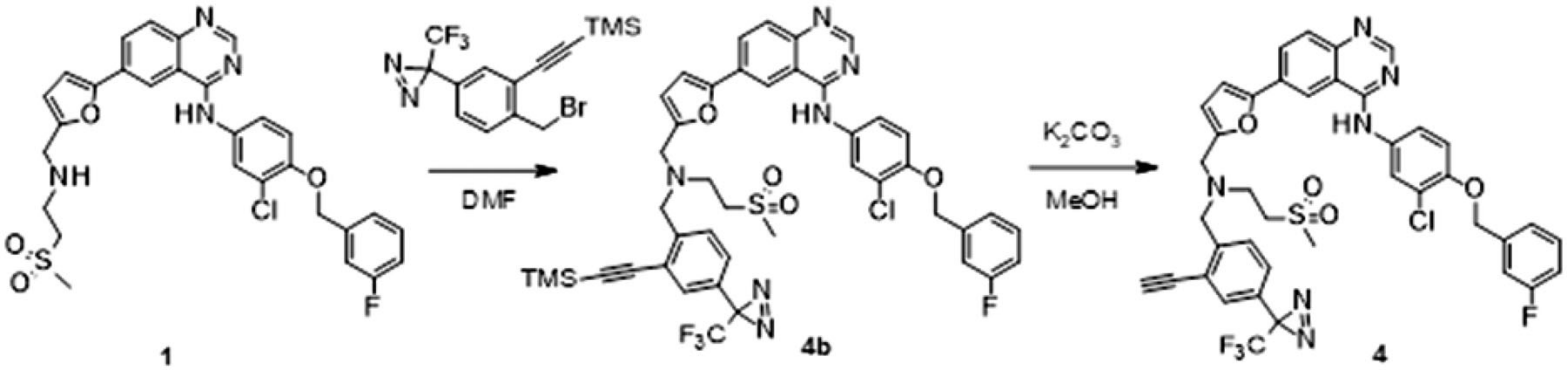
Synthesis of analogue **4**. Reagents and conditions: (a) NaH, DMF, rt; (b) K_2_CO_3_, MeOH, rt.

A solution of N-[3-chloro-4-[(3-fluorophenyl)methoxy]phenyl]-6-[5-[(2-methylsulfonylethylamino)methyl]-2-furyl]quinazolin-4-amine (**1**) (70 mg, 0.120 mmol) and sodium hydride, 60% disperse in mineral oil (9.6 mg, 0.240 mmol) in DMF (2 ml) was cooled to 0 °C. 2-[2-(bromomethyl)-5-[3-(trifluoromethyl)diazirin-3-yl]phenyl]ethynyl-trimethyl-silane (45 mg, 0.120 mmol) was then added (Scheme 5). The solution was allowed to stir at room temperature overnight. 2 ml of MeOH and potassium carbonate (49.7 mg, 0.360 mmol) were added. The solution was stirred at room temperature for seven hours. The reaction mixture was quenched with 10 ml of water and extracted with EtOAc (3 × 30 ml). The solvent was evaporated under reduced pressure. The sample was purified by flush chromatography using a BIOTAGE SP1 purification device and a 10 g normal phase silica SNAP column (DCM-EtOH solvent system with a gradient rising from 0–10% of EtOH in 15 CV). After evaporation of the solvent, 15 mg of N-[3-chloro-4-[(3-fluorophenyl)methoxy]phenyl]-6-[5-[[[2-ethynyl-4-[3-(trifluoromethyl)diazirin-3-yl]phenyl]methyl-(2-methylsulfonylethyl)amino]methyl]-2-furyl]quinazolin-4-amine (**4**) was isolated as a yellow solid (yield = 14.5%).

MS (Q-TOF MS): *m/z* calculated for C40H31ClF4N6O4S 803.1825 [M + H]^+^, and found to be 803.1851 [M + H]^+^.

Retention time: 8.13 min; Purity >93% (based on HPLC analysis at 230 nm).

^1^H NMR (400 MHz, DMSO-d_6_) *δ*/ppm: 9,84 (s, 1H), 8.70 (s, 1H), 8.57 (s, 1H), 8.13 (d, *J* = 8.81 Hz, 1H), 8.01 (d, *J* = 2.55 Hz, 1H), 7.70–7.84 (m, 3H), 7.42–7.51 (m, 1H), 7.24–7.36 (m, 5H), 7.11–7.22 (m, 1H), 7.02 (d, *J* = 3.24 Hz, 1H), 6.56 (d, *J* = 3.31 Hz, 1H), 5.28 (s, 2H), 4.58 (s, 1H), 3.93 (s, 2H), 3.83 (s, 2H), 3.45 (t, *J* = 6.82 Hz, 2H), 3.02 (s, 3H), 2.92–3.00 (m, 2H).

^13^C NMR (100 MHz, DMSO-d_6_) *δ*/ppm: 157.54, 154.26, 152.41, 152.04, 150.55, 149.76, 148.87, 144.45, 143.44, 139.63 (d, *J*_C-F_ = 7.37 Hz), 133.04, 130.54 (d, *J*_C-F_ = 8.84 Hz), 130.03, 129.82, 128.49, 128.47, 128.12, 126.76, 126.34, 124.23, 123.32, 122.41, 116.66, 115.28, 114.67 (d, *J*_C-F_ = 20.57 Hz), 114.31, 114.01 (d, *J*_C-F_ = 21.94 Hz) 111.87, 107.66, 87.10, 80.2, 69.38, 55.01, 51.09, 49.17, 46.63, 41.16.

#### Synthesis of 2-[2-(bromomethyl)-5-[3-(trifluoromethyl)diazirin-3-yl]phenyl]ethynyl-trimethyl-silane (5)

**Scheme 6.**
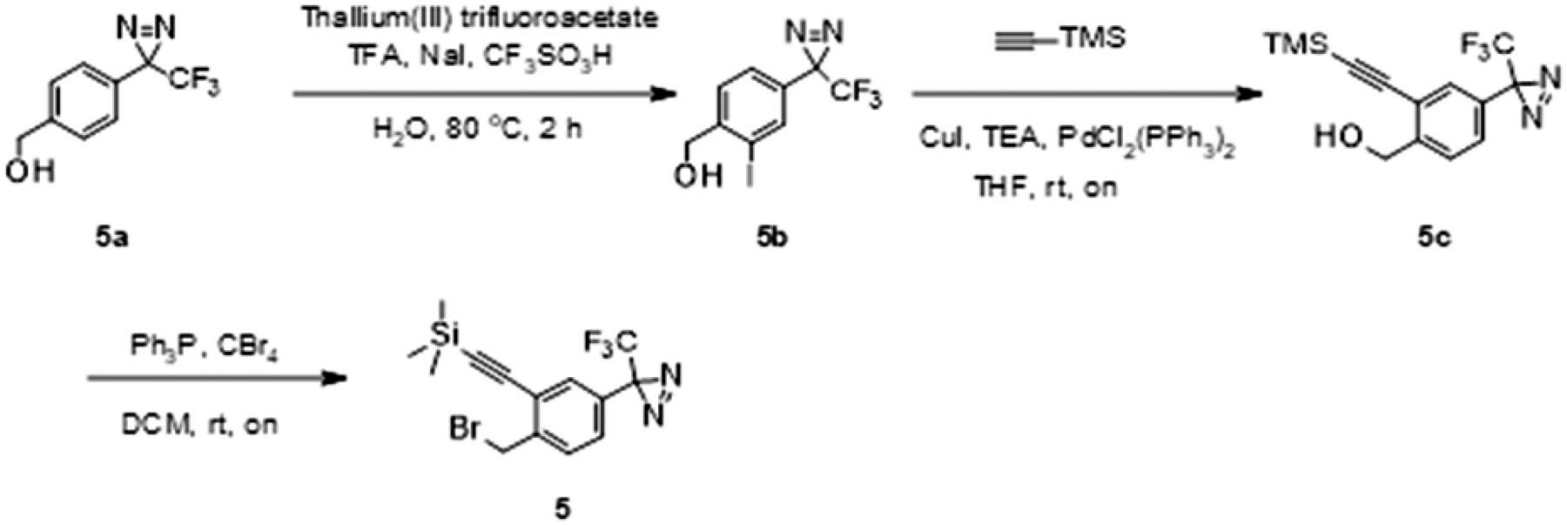
Synthesis of intermediate **5.** Reagents and conditions: (a) thallium(III) trifluoroacetate, TFA, NaI, CF_3_SO_3_H, H_2_O, 80 °C, 2h; (b) (trimethylsilyl)acetylene, CuI, TEA, PdCl_2_(PPh_3_)_2_, THF, rt, on; (c) Ph_3_P, CBr_4_, DCM, rt, on.

Thallium (III) trifluoroacetate (2.93 g, 0.0054 mmol) was dissolved in 5.5 ml of Trifluoroacetic acid. After addition of trifluoromethanesulfonic acid (1.43 ml, 0.0162 mmol), the white precipitate was dissolved by dropwise addition of water (0.22 ml) (Scheme 6). **5a** (410 mg, 0.0018 mmol) was added to this solution and the mixture was kept at 80 °C for 2 h. The reaction mixture was allowed to cool to room temperature and a solution of sodium iodide (4.05 g, 0.027 mmol) in 19 ml of water was added. After being stirred for 45 min in the dark, the elemental iodine that formed was reduced with sodium hydrogen sulphite. The solution was made alkaline with potassium hydroxide platelets. One volume of THF was added, and the yellow thallium(I)iodide precipitate was removed by filtration through Celite. The filtrate was extracted twice with Et_2_O, and the pooled organic phases were washed with water and dried over MgSO_4_. The solvent was evaporated under reduced pressure. The sample was purified by flush chromatography using a BIOTAGE SP1 purification device and a 25 g normal phase silica SNAP column (solvent system EtOAC-cyclohexane using gradient 0–20% of EtOAc in 15 CV). After evaporation of the solvent, 393.1 mg of product [2-Iodo-4–(3-trifluoromethyl-3H-diazirin-3-yl)-phenyl]-methanol (**5b**) was isolated as a white solid (yield = 64%).

^1^H NMR (400 MHz, DMSO-d_6_) *δ*/ppm: 7,60 (s, 1H), 7.58 (d, *J* = 8.10 Hz, 1H), 7.41 (d, *J* = 8.19 Hz, 1H), 5.62 (t, *J* = 5.56 Hz, 1H), 4.41 (d, *J* = 5.46 Hz, 2H).

^13^C NMR (100 MHz, DMSO-d_6_) *δ*/ppm: 146.64, 135.85, 127.91, 127.60, 126.51, 121.64 (q, *J*_C-F_ = 273.78 Hz), 97.21, 66.99, 27.24 (q, *J*_C-F_ = 39.87 Hz).

MS (Q-TOF): *m/z* calcd for C_9_H_6_F_3_IN_2_O 340.9404 [M–H]^−^, found 340.9532[M–H]^-^.

Bis(triphenylphosphine)palladium(II) dichloride (30.8 mg, 0.044 mmol), Copper(I) iodide (16.9 mg, 0.088 mmol), and Triethylamine (310.1 µL, 1.222 mmol) were added to a solution of **5b** (380 mg, 1.111 mmol) in THF under argon atmosphere. Ethynyltrimethylsilane (230.7 µL, 1.666 mmol) was then added dropwise using a syringe. The resulting solution was stirred at room temperature overnight. The reaction mixture was filtered through Celite and the solvent was removed by rotary evaporation. The residue was treated with water and extracted with ethyl ether. The combined organic layer was washed with brine and dried over magnesium sulphate. The solvent was then evaporated. The sample was purified by flush chromatography using BIOTAGE SP1 purification device and a 25 g normal phase silica SNAP column (solvent system cyclohexane-DCM using gradient 0–5% of DCM in 15 CV). After evaporation the of solvent, 241 mg of [4-[3-(trifluoromethyl)diazirin-3-yl]-2–(2-trimethylsilylethynyl)phenyl]methanol (**5c**) was isolated as a yellow solid, yield =69%.

^1^H NMR (400 MHz, DMSO-d_6_) *δ*/ppm: 7.65 (d, *J* = 8.22 Hz, 1H), 7.36 (d, *J* = 8.41 Hz, 1H), 7.22 (s, 1H), 5.45 (br.s, 1H), 4.62 (s, 2H), 0.24 (s, 9H).

^13^C NMR (100 MHz, DMSO-d_6_) *δ*/ppm: 146.97, 129.06, 127.05, 127.01, 125.94, 121.70 (q, *J*_C-F_ = 275.74 Hz), 120.31, 101.12, 100.77, 60.55, 27.73 (q, *J*_C-F_ = 39.81 Hz), −0.29 (3C).

Triphenylphosphine (176.2 mg, 0.679 mmol) was added slowly at 0 °C to a solution of **5c** (240 mg, 0.768 mmol). The mixture was stirred at room temperature overnight. Hexane was added to the reaction mixture and the precipitate was removed by filtration over Celite. The filtrate was evaporated *in vacuo*. The sample was purified on a BIOTAGE SP1 purification device, by chromatography, using a 10 g normal phase silica SNAP column and a DCM:EtOH solvent system (gradient rising from 0–5% of EtOH in 15 CV). The solvent from collected in fractions, and those of appropriate composition were evaporated. 237.8 mg of 2-[2-(bromomethyl)-5-[3-(trifluoromethyl)diazirin-3-yl]phenyl]ethynyl-trimethyl-silane (**5**) was isolated as a yellow oil, yield = 66.8%.

^1^H NMR (300 MHz, DMSO-d_6_) *δ*/ppm: 7.70 (d, *J* = 8.21 Hz, 1H), 7.36 (d, *J* = 8.22 Hz, 1H), 7.30 (s, 1H), 4.76 (s, 2H), 0.27 (s, 9H).

^13^C NMR (75 MHz, DMSO-d_6_) *δ*/ppm: 141.98, 130.96, 129.70, 128.20, 127.86, 123.36, 120.55 (q, *J*_C-F_ = 276.88 Hz), 101.70, 100.56, 31.19, 27.50 (q, *J*_C-F_ = 39.28 Hz), −0.45 (3 C).

### Biochemical experiments

#### Enzyme assay

Inhibition of lapatinib and lapatinib analogues to EGFR activity was determined at Eurofins DiscoverX Corporation using KINOMEscan technology.

#### A431 lysate preparation

A431 cells (ATCC) were grown in Dulbecco’s modified Eagle’s medium, supplemented with 10% foetal bovine serum. 1 × 108 of A431 cells were washed with PBS and lysed in 10 ml of lysis buffer (PBS/1% Triton X-100, supplemented with protease and phosphatase inhibitors (Roche), pH 7.4) on ice for 20 min. After centrifugation at 14,000 rpm, supernatants were stored at −20 °C until analysis.

#### Pull-down experiment with amino group

The experiment was performed as previously described1. 0.007, 0.035 and 0.11 μmol of lapatinib analogue was mixed with 25 μl of AffiGel 10 matrix.

#### Pull-down experiment with alkyne group

The alkyne analogue was immobilised to azide agarose resin using a Click and Go alkyne-tag enrichment kit (Jena Bioscience) according to manufacturer’s instructions. 25 μl of the resin was incubated with 0.05 and 0.2 μmol of the compound in DMSO. Incubation with cell lysate and elution of bound proteins were described previously.^20^

#### Pull-down experiment with dual tag group

The dual tag analogue was immobilised to azide agarose as described in the previous section. After incubation with A431 lysate for 3h at +4 °C, samples were illuminated with UV light (Spectroline CX-20 UV chamber) for 60 min. Resins were washed afterwards according to manufacturer’s instructions (Jena Bioscience) and trypsin digestion was carried out on the resins.

### Electrophoresis

Proteins eluted from matrices were separated by denaturing electrophoresis (Invitrogen, 12% gels) for 50 min at 170 V. Gels were stained with silver nitrate according to the manufacturer’s instructions (Sigma).

Protein spots were excised from 1-D gels into small pieces and subjected to in-gel digestion with trypsin according to the procedure.^21^
